# Platelet Additive Solutions as an Alternative Storage Medium of Apheresis Platelets to Reduce ABO Antibody Titer for ABO-Incompatibility Platelet Transfusion

**DOI:** 10.7759/cureus.44012

**Published:** 2023-08-23

**Authors:** Sumaiyah Adzahar, Mohd Nazri Hassan, Zefarina Zulkafli, Noor Haslina Mohd Noor, Marini Ramli, Shafini Mohamed Yusoff, Sien Tzi Lam, Rajesh Deshpande, Wan Zaidah Abdullah

**Affiliations:** 1 Department of Pathology, Faculty of Medicine, Universiti Sultan Zainal Abidin (UniSZA), Terengganu, MYS; 2 Department of Hematology, School of Medical Sciences, Universiti Sains Malaysia, Kota Bharu, MYS; 3 Fresenius, Kabi, Kuala Lumpur, MYS

**Keywords:** htr, platelet transfusion, antibody titer, apheresis platelet, pas

## Abstract

Introduction

Platelet additive solutions (PASs) are nutrient media commonly used to replace and reduce the need for storage plasma. They are an alternative medium to maintain high-quality platelets lasting longer on the shelf for about seven days. Platelets with high titer of ABO antibody can pose a hemolytic transfusion reaction (HTR) risk if units are given across the ABO barrier. The risk of complication is greater when group O platelet is released to non-group O patients. The PAS has been known as a safe medium, where the titer of ABO antibodies is expected to be diluted. In this study, we compared the anti-A and anti-B antibody titers of apheresis platelets in PAS and non-PAS (plasma) as the suspending media.

Methods

A total of 20 apheresis platelet donors were selected, with seven from blood group A, eight from blood group B, and five from blood group O. The platelets were collected using an Amicus cell separator. They were suspended in PAS and plasma before being stored at a temperature range of 22-24^º ^C. Anti-A (blood group B and O) and Anti-B (blood group A and O) antibody titers were measured and compared between the two suspending media. Wilcoxon signed-rank test is used for statistical analysis, and a p-value <0.05 is considered significant.

Results

The median titer of the anti-A antibody of apheresis platelets showed a significant difference between suspended in PAS (2.50) and plasma (4.00), p=0.002. Similar findings were also seen with the median titer of the anti-B antibody of apheresis platelet, in which it showed a significant difference between suspended in PAS (2.00) and plasma (4.00), p=0.004. It was observed that there was a significant reduction in both anti-A and anti-B antibody titers in the PAS as compared to the plasma group.

Conclusion

The decrease in ABO antibody titer in apheresis platelets stored with PAS can be beneficial for patients. This reduces the risk of HTRs if ABO-incompatible platelet units need to be issued. Thus, using PAS as a storage medium significantly improves platelet inventory management without compromising patient safety.

## Introduction

Platelet additive solutions (PASs) are electrolyte solutions used for platelet storage. Various types of PASs (e.g., PAS-I, PAS-II, and PAS-III) can be used for apheresis and buffy coat platelets [1]. PAS contains acetate, citrate, phosphate, sodium, potassium, magnesium, and glucose [2]. PASs have the advantage of removing more ABO-incompatible plasma, which in turn reduces the incidence of acute hemolytic transfusion reactions (HTRs) [3], as well as increases post-transfusion platelet recovery [4] in ABO-incompatible platelet transfusion. Additional benefits of PAS have been proposed to reduce the other adverse transfusion reactions, for example, transfusion-associated with circulatory overload and transfusion-related acute lung injury. However, definitive evidence remains insufficient [5]. PAS is not typically associated with causing allergic or anaphylactic transfusion reactions as it contains no proteins or components that would normally trigger an allergic or anaphylactic response.

Acute HTRs typically occur when the transfused plasma has a high titer of ABO antibodies that are incompatible with the recipient's blood type [5]. Platelets express ABO antigens on their surface and are present in the donor's plasma. For example, an individual who is a group O platelet donor does not express any A or B antigens on their platelets. However, their plasma has high levels of anti-A, anti-B, and anti-AB antibodies. These antibodies can cause the destruction of red blood cells in non-group O recipients, leading to HTRs [6]. Reducing the plasma in the platelet units has been shown to reduce anti-A and anti-B titers. Hence, using PAS is an alternative method for plasma reduction because much of the plasma can be replaced by it [7]. Thus, this study aimed to compare the level of anti-A and anti-B antibody titers in PAS and plasma (non-PAS) of apheresis platelets.

## Materials and methods

Study participants

The present study was an interventional study conducted from February 2019 to June 2019 in the Transfusion Medicine Unit, Hospital Universiti Sains Malaysia (USM). About 20 apheresis platelet donors who met the criteria for apheresis platelet donation according to Malaysia's National Blood Center guidelines were included. Donors who did not meet the usual requirements for an acceptable donation, for example, on medication (anticoagulant) and had chronic diseases (diabetes mellitus, hypertension, coronary artery disease, and renal disease), were excluded. The study was approved by the Institutional Human Research Ethics Committee (USM/JEPeM/18100593) and carried out following the Declaration of Helsinki. Written consent was obtained from all voluntary apheresis donors involved in the study.

Apheresis platelets preparation

Apheresis platelets were collected using Amicus cell separator version 3.2 (Fresenius Kabi, Germany). This process separates platelets from whole blood, concentrates them, and returns the remaining blood components to the donor. About 50 mL of platelets were separated from the collection bag and placed into a satellite bag for the study. The bag was then divided equally into two parts. One part was stored in plasma (non-PAS), while the other was stored in PAS (InterSol, Fenwal) fortified plasma with a ratio of 65:35 (PAS: plasma). The apheresis platelets were stored for seven days under standard conditions at a temperature range of 20-24ºC with continuous agitation.

Sampling and titration procedure

Sampling was done by stripping the tubing of the pedi-pack using sampling site couplers. The platelet was then collected into a plain tube (as the specimen was already in an anticoagulated form). Anti-A and anti-B antibody titers were measured on day 1 of the storage period. Anti-A titer was measured in 12 samples of apheresis platelets (seven blood group B and five blood group O); anti-B titers were measured in 13 samples (eight blood group A and five blood group O) for both PAS and plasma groups.

Anti-A and anti-B titers were determined by serological testing using a gel card method (CAT- Bio-rad). Serial dilutions were made from group A, B, and O apheresis platelets in 0.9% saline, using a calibrated pipette, ranging from 1:1 to 1:128 of PAS and plasma groups. Type A1 and B reverse typing cells (Bio-rad red cells reagent) were used to determine the anti-A and anti-B antibody titers. The reaction of the titer was read after the gel card was incubated at 37°C for 15 minutes and centrifuged for 10 minutes. The last dilution with a positive reaction of 1+ was considered the highest titer for the respected ABO antibody. The same individual performed all testing to avoid inter-examiner variations during testing.

Statistical analysis

The data were analyzed using a statistical package for the social sciences (SPSS) software version 26.0 (IBM Corp., Armonk, NY). The Wilcoxon Signed Ranks test was used to compare the anti-A and anti-B antibody titers between the suspended media and reported as a median and inter-quartile range (IQR). The p-value <0.05 was considered statistically significant.

## Results

Anti-A titers in PAS and plasma ranged from 1:1 to 1:32 (median titer = 2.50 (between titer 1: 2 to 1:4)) and 1:2 to 1:128 (median titer = 4.00 (at titer 1:8), respectively. Meanwhile, anti-B titers in PAS and plasma ranged from 1:1 to 1:128 (median titer = 2.00 (at titer 1:2), and 1:2 to 1:128 (median titer = 4.00 (at titer 1:8)), respectively (Table 1).

**Table 1:** Anti-A and anti-B titers stored in PAS and plasma a=measured in group B and O apheresis platelet; b=measured in group A and O apheresis platelet PAS - Platelet additive solution

Titer	Anti-A^a^ (n=12)		Anti-B^b^ (n=13)	
	PAS, n (%)	Plasma, n (%)	PAS, n (%)	Plasma, n (%)
1:1	1	0	2	0
1:2	3	1	2	1
1:4	2	1	4	5
1:8	2	2	1	0
1:16	2	4	0	3
1:32	2	3	2	0
1: 64	0	0	1	1
1:128	0	1	1	3
Median	2.5 (Between titer 1:2 to 1:4)	4.0 (At titer 1:8)	2.0 (At titer 1:2)	4.0 (At titer 1:8)
Range	1:1 to 1:32	1.2 to 1:128	1:1 to 128	1:2 to 1:128

The median titer of anti-A antibody showed a significant difference between PAS and plasma (p=0.002). Similar findings were also seen with the median titer of the anti-B antibody, which showed a significant difference between PAS and plasma (p=0.004), respectively (Table 2).

**Table 2:** Comparison of anti-A and anti-B in PAS and plasma Statistical analysis using Wilcoxon Signed Ranks test PAS - Platelet additive solution

Comparison	PAS median (IQR)	Plasma median (IQR)	Z-statistics	P-value
Anti-A	2.50 (3.00)	4.00 (2.00)	-3.03	0.002
Anti-B	2.00 (4.00)	4.00 (4.50)	-2.89	0.004

## Discussion

Platelet transfusion is clinically indicated as part of treatment or prophylaxis in patients with platelet-related disorders. Platelet concentrates (PC) are indicated therapeutically to treat bleeding manifestations in patients with thrombocytopenia or qualitative functional defects (inherited or acquired). PC may also be administered prophylactically to prevent bleeding when the platelet count is below the acceptable limit, such as before a surgical procedure [8].

In platelet transfusion, there are two types of ABO incompatibility [6]. The first is major incompatibility, which occurs when the recipient's plasma contains antibodies against the donor's ABO antigen on the platelets. This can quickly destroy the transfused platelets, causing poor post-transfusion platelet recovery. The second type is minor ABO-incompatibility, which occurs when the donor's plasma has antibodies against the recipient's ABO antigen on RBC [6-9].

Many institutions nowadays accept the practice of transfusing ABO-incompatible platelets. According to AABB’s Standards for Blood Banks and Transfusion Service, there must be a policy regarding the transfusion of blood components that can have a significant amount of incompatible ABO antibodies. It is possible for platelet transfusions to contain significant amounts of incompatible ABO antibodies or unexpected red cell antibodies, which can result in clinical relevance [10].

Reducing the amount of plasma in platelet products has been shown to reduce some transfusion reactions, including acute HTRs in minor incompatibility platelet transfusion. Previous studies showed that PAS is an alternative method for plasma reduction because two-thirds of plasma is replaced by PAS [7]. Weisberg et al. suggested using PAS to prevent acute HTRs caused by minor ABO-incompatible platelet transfusion [11]. Our study supports this recommendation as we have observed a significant reduction in ABO antibodies, specifically for both anti-A and anti-B antibodies in the PAS group. This is due to the 65% plasma replacement with PAS causing a dilution effect [12].

Furthermore, platelets with high titers of ABO antibodies can pose a risk of hemolysis if units are given across the ABO barrier. The risk is greater when group O platelet is released to non-group O patients [13]. The threshold for critical titers depends on the methodology used; however, there is a lack of past literature that clearly defines clinically critical titer levels. Based on the work of different researchers, most blood banks follow a cutoff ranging from 128 to 250 for defining high-titer units [14]. In our study, when 20 donors were tested, we discovered that only one apheresis platelet component in PAS exceeded our clinically applied threshold of titer 128. This result aligns with a similar finding reported by Tynuv et al. [10]. The practical implications of this finding are significant for the clinical application of PAS as a platelet's suspending media as it improved the management of out-of-group transfusion of platelet components.

Few studies have shown that platelets suspended in plasma from blood donors tend to have higher levels of anti-B antibodies compared to levels of anti-A antibodies [15]. Another study by Kavallierou et al. found that platelets stored in plasma from group O donors had higher levels of anti-A antibodies compared to anti-B antibodies [6]. This aligns with what has been documented in the literature regarding instances of HTRs that occur due to the passive transfer of anti-A and anti-B antibodies [16]. Our study revealed that both anti-A and anti-B antibody levels were significantly lower in PAS compared to plasma. This reduction in titer could benefit patients by lowering the risk of hemolysis if incompatible platelet units are used.

Overall, using PAS as a storage medium for apheresis platelets can effectively decrease ABO-incompatible antibody levels and improve inventory management, resulting in less waste. However, this study has a few limitations. First, a small sample size, as only 20 apheresis donors were available for this study. Thus, the reliability of PAS effects cannot be predicted. As a recommendation for future studies, the sample size collection should be made in larger collection centers such as the National Blood Center, where larger platelet apheresis donation is available. Second, detection methods for ABO antibody titers may vary across laboratories, and the results are expressed depending on the method used. There is still an issue with the lack of standardization in performing titers and the absence of an international consensus on what qualifies as a critical titer. Lastly, only in-vitro tests were performed, and their clinical impact on clinical platelet transfusion is under discussion. The precise correlation between in-vitro assays and in-vivo platelet recovery is yet to be established.

## Conclusions

Although the risk of hemolysis from passively transfused anti-A and anti-B is low, it is still present. Therefore, it is recommended to consider apheresis platelets suspended in PAS to decrease the risk of HTRs as well as the expected reduction in the incidence of hemolysis. Furthermore, assessment of platelet function in PAS will guide the clinical benefit of PAS medium for apheresis platelets.
